# Sight the Mite: A Meta-Analysis on the Diagnosis of Scabies

**DOI:** 10.7759/cureus.34390

**Published:** 2023-01-30

**Authors:** Qaas Shoukat, Ahraz Rizvi, Waseem Wahood, Skyler Coetzee, Algevis Wrench

**Affiliations:** 1 College of Medicine, Dr. Kiran C. Patel College of Allopathic Medicine, Nova Southeastern University, Davie, USA; 2 Medicine, Dr. Kiran C. Patel College of Allopathic Medicine, Nova Southeastern University, Davie, USA; 3 Microbiology, Nova Southeastern University, Fort Lauderdale, USA

**Keywords:** diagnostic test accuracy, diagnostic testing, parasitic disease, infectious and parasitic diseases, systematic review and meta analysis, systematic review and meta-analysis, neglected (re)emergent tropical disease, neglected disease, sarcoptes scabiei var. hominis, diagnosis of scabies

## Abstract

This meta-analysis was performed to assess the efficacy of the diagnostic tests for scabies infections that are currently in wide use. Scabies is most commonly diagnosed through clinical presentations; however, due to the wide array of symptoms, diagnosis is difficult. The most commonly used diagnostic test is skin scraping. However, this test relies on correctly selecting the site of mite infection for sampling. Due to the mobile nature of a live parasitic infection, the mite can often be missed based on its current location within the skin. The goal of this paper is to determine if a gold standard confirmatory test exists for the diagnosis of scabies by comparing Skin Scraping, Adhesive Tape, Dermoscopy, and PCR tests. Medline, PubMed, and Neglected Tropical Diseases databases were utilized in a literature review. Eligible papers were papers published in or after the year 2000, published in the English language, and mainly focused on the diagnosis of scabies. At the time of this meta-analysis, scabies is mostly diagnosed through a correlation of clinical symptoms in conjunction with diagnostic tests such as dermoscopy (sensitivity: 43.47%, specificity: 84.41%), adhesive tape tests (sensitivity: 69.56%, specificity: 100%) and PCR antigen detection (37.9% sensitivity, specificity: 100%). Due to a scarcity of data in the literature, the diagnostic efficacy of other diagnostic tests is difficult to assess. Overall, the efficacies of the tests analyzed vary depending on how similar scabies is to other skin disorders, how challenging it is to get a usable sample and the price and accessibility of essential tools. There is a need for standardized national diagnostic criteria to increase the diagnostic sensitivity of scabies infection.

## Introduction and background

Scabies refers to the dermatologic infection caused by the Sarcoptes scabiei var. hominis mites [1]. It is a parasitic disease that is highly contagious and is marked by severe pruritis and a variety of cutaneous symptoms. With an estimated global incidence of over 200 million cases per year and a prevalence of 1.725 million, and an incidence of 5.154 million in the United States between 1990 and 2017, scabies infection bears a heavy disease burden [2]. Scabies primarily infects infants, children, and adolescents, and has a higher prevalence in low-income settings and tropical areas, although infestations are common in all countries regardless of the development level. In developed countries, outbreaks typically occur in densely populated settings that involve close contacts, such as prisons, nursing homes, and childcare facilities [1]. Scabies has an incubation period of four to six weeks [3]. A delayed-type hypersensitivity to mites and their byproducts leads to characteristic pruritus and skin lesions. In some patients, but not all, burrows may be seen. Patients who have previously been exposed typically experience symptoms considerably more quickly (within hours to days). The infestation does not provide full immunity or protection against future exposure; however, the complete immune response is poorly understood [4]. Recurrent episodes are thus frequent in areas of high transmission, particularly in children [5,6]. Common scabies is the term used to describe this set of symptoms and indications (also described as classical, typical, ordinary, standard, usual, or normal scabies).

Scabies is most commonly diagnosed based on clinical presentation. Due to the wide constellation of signs and symptoms, however, a clinical diagnosis may be difficult to make. Approaches to diagnosing scabies include the scraping of skin lesions and subsequent microscopic examination to look for mites or mite products; the adhesive skin tape test, which involves applying adhesive tape to a suspected lesion and then transferring the tape to a slide that is examined under a microscope; and dermatoscopy. While these diagnostic techniques are currently in widespread use, other techniques currently under investigation are blood tests that involve the use of PCR or ELISA and other more advanced technological approaches such as reflectance confocal microscopy, epiluminescence microscopy, and video dermatoscopy. The current challenge to the effective diagnosis of scabies is obtaining a sample from a patient that confirms a scabies diagnosis. The current techniques of skin scrapings, adhesive tape tests, and dermoscopy rely on correctly choosing the site of mite infestation but also visualizing the mite and/or its products in that sample. Due to the nature of a live parasitic infection, however, the mite is mobile and can often be missed based on its current location within the skin. The techniques currently under investigation either require further study to evaluate their effectiveness or rely on sophisticated technology that may not be readily available in most settings. The goal of this paper is to perform a meta-analysis of the diagnostic techniques currently in use and/or under investigation and focuses on Skin Scraping, Adhesive Tape, Dermoscopy, and PCR. Very few studies evaluating the sensitivities and specificities of these tests exist in the literature, and given scabies’ status as a neglected tropical disease, it is essential to quantify the efficacy of the current diagnostic protocol and promote research into more effective methods. Note some of the contents of this paper were presented as part of a poster titled: “The Mighty Mite: A Literature Review on the Diagnosis of Scabies” as part of the HCA-NSU MD Research Day in 2020 [7].

Methods

**Figure 1:**
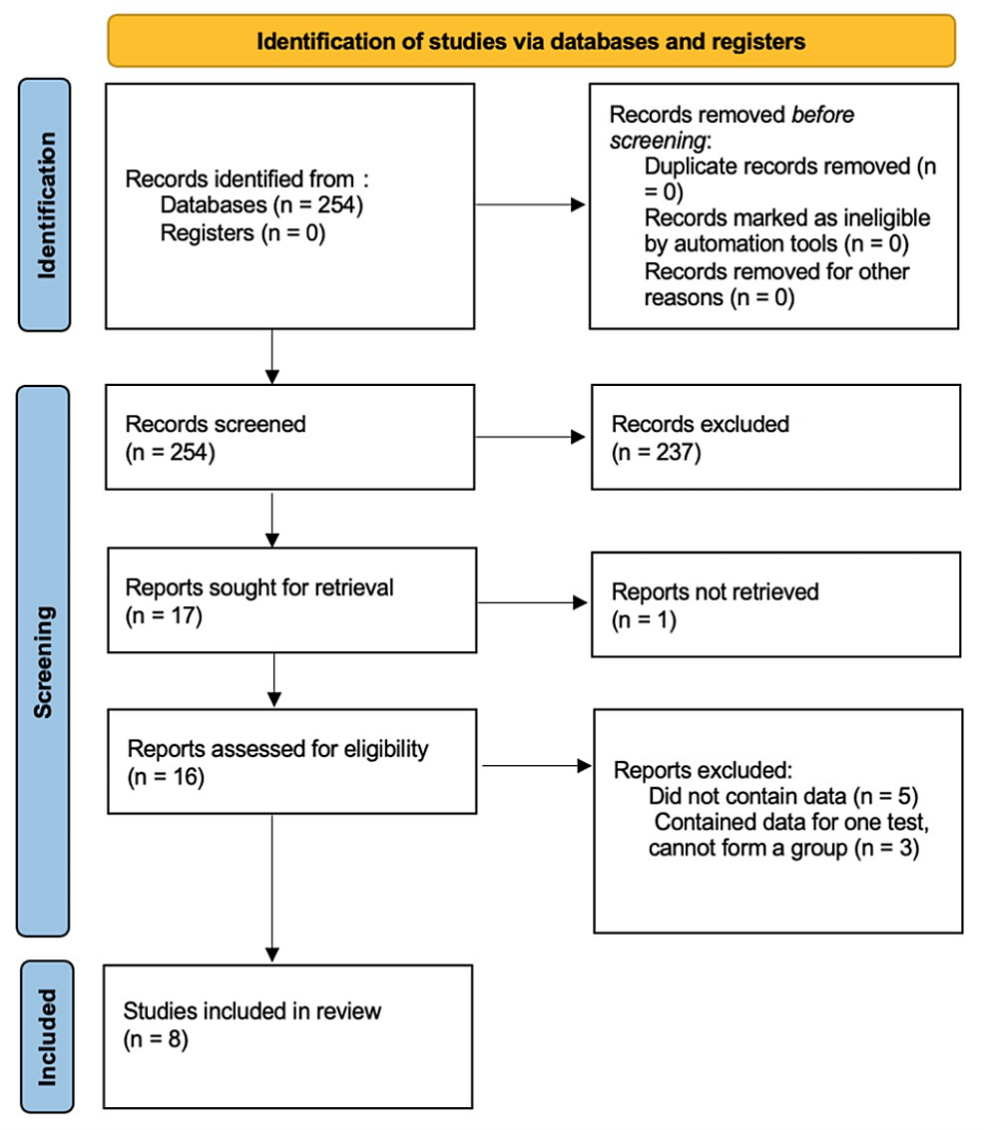
PRISMA Search Strategy Flow Chart of the Present Systematic Review and Meta-Analysis

This systematic review and meta-analysis were conducted using the PICO (Patient, Intervention, Comparator, and Outcome) format and followed the framework outlined in the PRISMA (Preferred Reporting Items for Systematic Reviews and Meta-Analyses) guidelines (Figure 1) [8]. The PICO format for this current study was as follows: (P)atients with scabies, (I)ntervention and (C)omparison of skin scraping, adhesive tape, dermoscopy, and PCR as diagnostic tools, and O(utcome) of sensitivity and specificity. 

*Search Strategy* A comprehensive search of only English articles published on PubMed from the first archived date to July 27^th^, 2020, was conducted. The search strategy was designed and conducted by two authors (Q.S. and A.R.) and reviewed by a third author (W.W.). Keyword searches included “(Sarcoptes Scabiei) AND ((Sensitivity) OR (Specificity) OR (Diagnostic))”.

*Study Selection Criteria* Studies were included for review if they met the following criteria: studies with human subjects, Sarcoptes scabiei var. Hominis (human scabies), and with sensitivity and/or specificity data. Studies were excluded based on the following pre-defined criteria: Case studies, studies with non-human subjects, and studies focusing on non-human scabies.

*Quality Assessment* The GRADE (Grading of Recommendations, Assessment, Development, and Evaluations) approach [9] was used to grade the outcomes' quality, and the modified Newcastle-Ottawa Scale [10] was used to grade the studies' quality of evidence.

*Data Extraction* According to the aforementioned inclusion and exclusion criteria, eligible studies were pooled, and three reviewers (Q.S., A.R., and W.W.) gathered data from papers, tables, and figures. The accuracy of the data entries was confirmed by the third reviewer (W.W). Study author, year of publication, diagnostic test type, sample size, method of diagnosis verification, true positives, true negatives, false positives, false negatives, specificity, and sensitivity were among the information gathered (Table 1). The tests that were used were dermoscopy, adhesive tape, skin scraping, and PCR.

**Table 1:** Characteristics of Studies PCR = Polymerase Chain Reaction

First Author's Last Name	Year	Country	Total Sample Size	% (or #) Female	% (or #) Male	Average Age	Test Name	How was Scabies Confirmed?
Latif et al., 2018 [11]	2018	Egypt	100	40%	60%	-	Skin scraping, Adhesive Tape, Dermoscopy	Clinical Diagnosis
Walter et al., 2011 [12]	2011	Germany	113	69	44	14	Skin scraping, Adhesive Tape, Dermoscopy	Clinical Diagnosis
Delaunay et al., 2020 [13]	2020	France	164	86	78	10	PCR	Dermoscopy/Clinical Diagnosis
Wong et al., 2015 [14]	2015	Hong Kong	100	18	11	87	PCR	Microscopy
Dupuy et al., 2006 [15]	2006	France	245	-	-	-	Dermoscopy	Skin scraping
Bae et al., 2020 [16]	2020	Republic of Korea	47	-	57%	-	PCR	Microscopy
Zorbozan et al., 2020 [17]	2020	Turkey	42	19	23	39	Skin scraping	1 or more burrows on video dermatoscopy
Hahm et al., 2018 [18]	2018	Korea	63	36	27	59.41	PCR	Microscopy

*Statistical Analysis* The results of a meta-analysis on the specificity and sensitivity of the diagnostic tests are shown as effect sizes (ES) along with their corresponding 95% confidence intervals (CIs). The DerSimonian and Laird approach [19] with a random effects model was employed. The differences in the groups represent the statistical variation in the results of the three groups. STATA 16.0 and the "metan" package were used for the statistical analysis (StataCorp. 2019. Stata Statistical Software: Release 16. College Station, TX: StataCorp LLC.) [20]. A p-value of < 0.05 was considered significant.

## Review

Results

Characteristics of Studies

The total number of studies included was eight; four involved skin scraping, two involved adhesive tapes, three involved dermoscopy, and four involved PCR [11-18]. In total, 1544 patients were included in this meta-analysis. The mean age in all studies was 41.8. The percentage of females ranged from 19% to 86% (Table 1).

**Figure 2:**
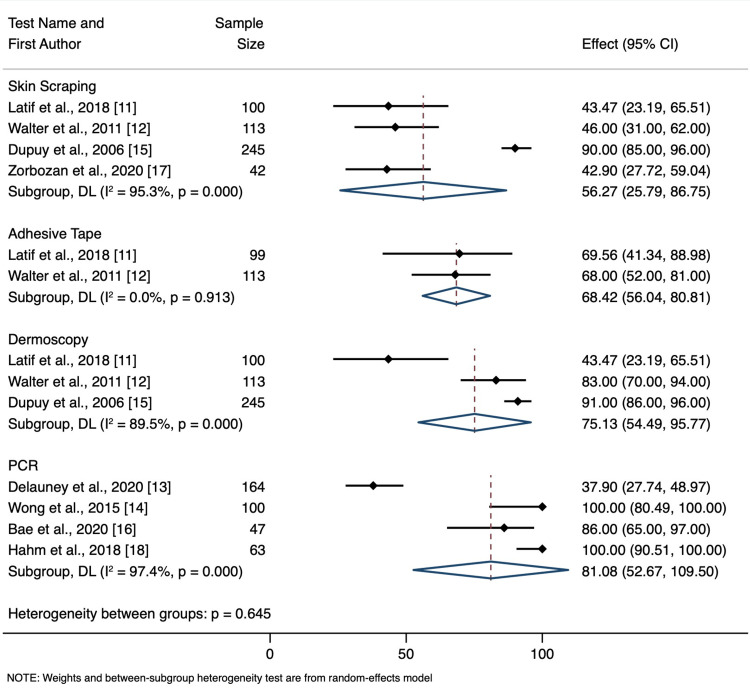
Sensitivity Forest Plot CI = Confidence Interval, DL = DerSimonian and Laird Random Effect Model, p = p-value, I^2 ^= percent heterogeneity Latif et al., 2018 [11], Walter et al., 2011 [12], Delaunay et al., 2020 [13], Wong et al., 2015 [14], Dupuy et al., 2006 [15], Bae et al., 2020 [16], Zorbozan et al., 2020 [17], Hahm et al., 2018 [18]

*Sensitivity* The overall sensitivity between tests was not statistically significant (p=0.645). Skin scraping had an average sensitivity of 56.3% (95% CI: 25.8 to 86.8); adhesive tape had an average sensitivity of 68.4% (95% CI: 56.0 to 80.8); dermoscopy had an average sensitivity of 75.1% (95% CI: 54.5 to 95.8); PCR had an average sensitivity of 81.1% (95% CI: 52.7 to 100). This can be seen in Figure 2.

**Figure 3:**
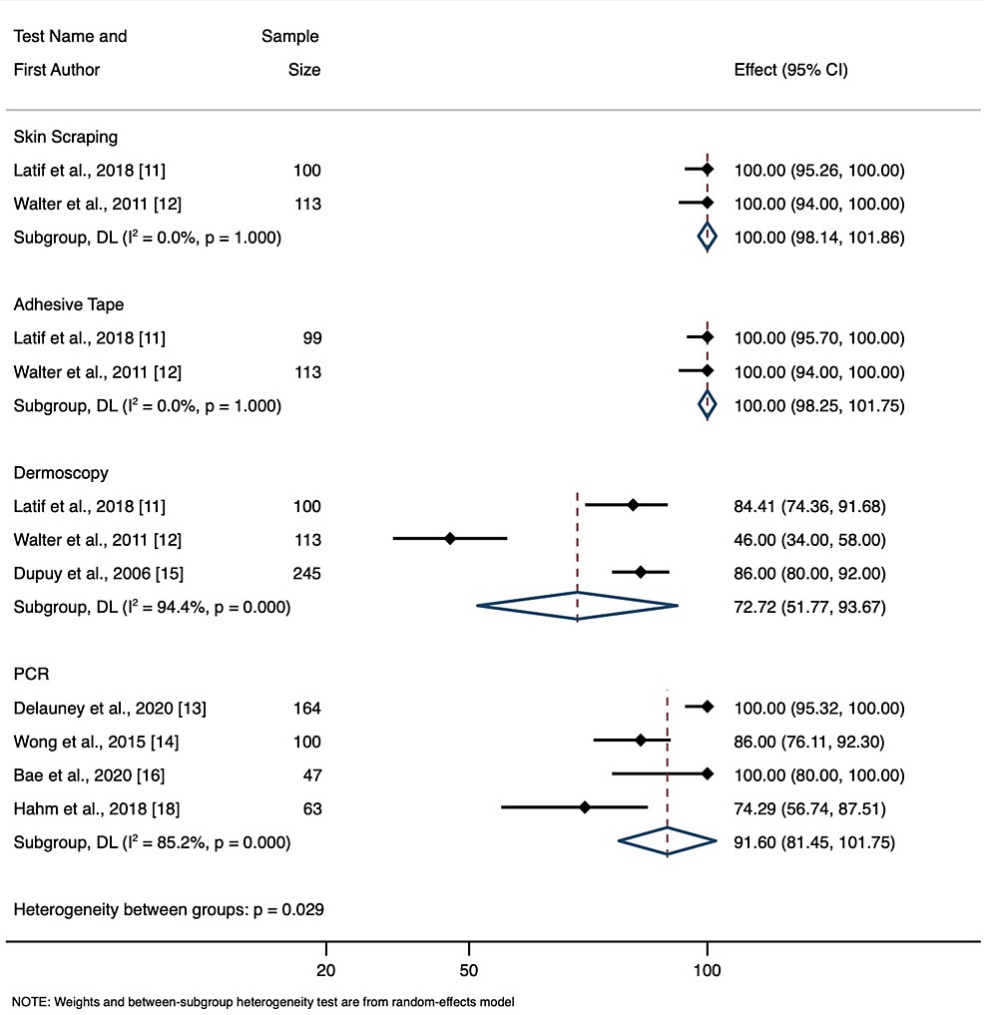
Specificity Forest Plot CI = Confidence Interval, DL = DerSimonian and Laird Random Effect Model, p = p-value, I^2^ = percent heterogeneity Latif et al., 2018 [11], Walter et al., 2011 [12], Delaunay et al., 2020 [13], Wong et al., 2015 [14], Dupuy et al., 2006 [15], Bae et al., 2020 [16], Zorbozan et al., 2020 [17], Hahm et al., 2018 [18]

*Specificity* The overall specificity between tests was statistically significant (p=0.029). Skin scraping had an average specificity of 100% (95% CI: 98.1 to 100.0); adhesive tape had an average sensitivity of 100% (95% CI: 98.3 to 100.0); dermoscopy had an average sensitivity of 72.7% (95% CI: 51.8 to 93.7); PCR had an average sensitivity of 91.6% (95% CI: 81.5 to 100.0). This can be seen in Figure 3.

*Quality of Evidence* Based on the GRADE approach, the certainty assessment was found to be low for sensitivity and moderate for specificity (Table 2). This study is an indirect meta-analysis; therefore, indirectness was assessed as a serious bias. Additionally, sensitivity results had wide confidence intervals, indicating a serious imprecision. The quality of evidence was found to be satisfactory across all studies via the Newcastle Ottawa Scale (Table 3).

**Table 2:** GRADE Assessment NOS = Number of Studies, ROB/Inc. = Risk of Bias/Inconsistency, Ind. = Indirectness, Imp. = Imprecision, OC = Other Considerations, SS = Skin Scraping, AT = Adhesive Tape, DS = Dermoscopy, PCR = Polymerase Chain Reaction, CI = Confidence Interval

Certainty assessment						# of patients				Relative Effect (95% CI)	Certainty
NOS	Study design	ROB/Inc.	Ind.	Imp.	OC	SS	AT	DS	PCR		
Sensitivity											
8	observational	Not Serious	Serious	Serious	none	500	212	458	285	SS: 56.3% (95% CI: 25.8 to 86.8)	⨁⨁◯◯ LOW
										AT: 68.4% (95% CI: 56.0 to 80.8)	
										DS: 75.1% (95% CI: 54.5 to 95.8)	
										PCR: 81.1% (95% CI: 52.7 to 100)	
Specificity											
7	observational	Not Serious	Serious	Not Serious	none	213	212	458	374	SS: 100% (95% CI: 98.1 to 100.0)	⨁⨁⨁◯ MODERATE
										AT: 100% (95% CI: 98.3 to 100.0)	
										DS: 72.7% (95% CI: 51.8 to 93.7)	
										PCR: 91.6% (95% CI: 81.5 to 100.0)	

**Table 3:** Newcastle Ottawa Table ^a^Studies that do not fulfill these criteria had a much lower average age than the rest of the studies. ^b^Studies that do not fulfill these criteria used another test to ascertain exposure rather than the gold standard.

Author Year	Representativeness of the Cohort^a^	Ascertainment of Exposure^b^	Outcome of Interest	Assessment of Outcome	Adequate Follow-up Duration
Latif et al., 2018 [11]	*	*	*	*	*
Walter et al., 2011 [12]	-	*	*	*	*
Delaunay et al., 2020 [13]	-	*	*	*	*
Wong et al., 2015 [14]	*	*	*	*	*
Dupuy et al., 2006 [15]	*	-	*	*	*
Bae et al., 2020 [16]	*	*	*	*	*
Zorbozan et al., 2020 [17]	*	*	*	*	*
Hahm et al., 2018 [18]	*	*	*	*	*

Discussion

In the United States, there is a lack of official guidelines for the diagnosis of scabies infections. This leads to reliance on physician preference in screening and diagnosis. Clear, consistent diagnostic criteria may increase both a clinical suspicion of scabies infection and diagnostic sensitivity and reduce delays in diagnosis. Our results indicate that there was no difference in the sensitivity of detecting scabies between skin scraping, adhesive tape, dermoscopy, or PCR. Overall specificity was 100% for both skin scraping and adhesive tape, while it was 91.6% for PCR and 72.7% for dermoscopy. Although the adhesive tape test and the skin scraping test have the highest specificity, they require the presence of ticks in the area of the skin being tested, which could provide a false negative result. The skin scraping and adhesive tape tests have a high specificity because the direct visualization of a mite, mite eggs, or mite droppings is irrefutable evidence of scabies infestation. While these features are clearly identifiable and highly specific for infestation, they also have low sensitivity because there is a possibility that they are absent in the area that is sampled. This would lead to false negative results. The high specificity of PCR could be explained by the fact that if parasite loads are high enough to be detectable on testing, a true infection by scabies mites is likely. The relatively low specificity of dermoscopy could be explained by the relatively nonspecific findings seen in scabies infestation. These findings include erythematous, pruritic papules, excoriations, and linear burrows in the classical distribution [21]. There are a few limitations of scabies diagnostic research that should be considered in this study. There is very little data published regarding approaches to diagnosing scabies, likely due to their low incidence in the United States. The lack of official diagnostic guidelines in the United States leads to a reliance on the discretion of individual physicians. Clear diagnostic criteria would help refine clinical discretion in pursuing further confirmatory testing. Notably, there are a limited number of studies that assess the specificity and sensitivity of the various diagnostic methods, and those that do often have small sample sizes. This makes application to the clinical setting challenging. To improve the statistical power and external validity of the data available, a meta-analysis was performed. The efficacies of the various diagnostic techniques differ based on several factors. The difficulty of obtaining samples is particularly troublesome because, in some techniques, the tested area must contain the mite or its associated elements. If findings are not observed in the tested area, it does not rule out the presence of scabies in non-tested areas [22]. For tests such as Serum ELISA detection and PCR Antigen Detection, the similarity of scabies to other species leads to cross-reactivity and relatively low sensitivity and specificity. Methods such as Reflectance Confocal Microscopy and Epiluminescence Microscopy may not be feasible diagnostic tools in facilities without the means to acquire the technology. Additionally, the specificity of some of these methods is often 100% by default since the presence/absence of the observed result is irrefutable evidence of scabies infection, such as the visualization of mite/mite eggs/mite droppings. With these findings, we infer no procedure can be deemed a gold standard for the diagnosis of scabies. For this reason, more investigation is necessary to determine if any of the diagnostic modalities are superior in sensitivity to the clinical features that are currently utilized to diagnose scabies infection.

*Strengths and Limitations* Regarding this particular study, there were several notable limitations. First, due to limitations in institutional access, only PubMed was used for our database search. Second, some studies used multiple diagnostic tests to verify the diagnosis of scabies. The use of additional studies makes it difficult to ascertain the utility of a single diagnostic test, which was the measure our study investigated. Lastly, an indirect comparison was performed for this meta-analysis, which is a lower level of evidence compared to a direct meta-analysis with all two-arm studies.

## Conclusions

Scabies is a widely underdiagnosed infection in the United States. The relatively low prevalence and lack of a gold standard diagnostic tool contribute to underdiagnosis and increased disease burden. In this paper, we investigated several tools most commonly used to verify scabies infection, including skin scraping, tape test, PCR, dermoscopy, and epiluminescence. We found that there was no significant difference in the sensitivity of these diagnostic tests. However, the studies varied in their specificity for confirming scabies infection. We conclude that due to the highly infective nature of scabies, as well as the severity of symptoms, maintaining high clinical suspicion is critical to accurate and timely diagnosis. Secondary testing may be of utility if the diagnosis is uncertain. More studies are necessary to evaluate whether any test(s) can be deemed a gold standard.
